# A Cable-Driven Three-DOF Wrist Rehabilitation Exoskeleton With Improved Performance

**DOI:** 10.3389/fnbot.2021.664062

**Published:** 2021-04-08

**Authors:** Ke Shi, Aiguo Song, Ye Li, Huijun Li, Dapeng Chen, Lifeng Zhu

**Affiliations:** School of Instrument Science and Engineering, Southeast University, Nanjing, China

**Keywords:** cable-driven robot, rehabilitation robot, mechanism design, distributed drive system, human-robot interaction

## Abstract

This paper developed a cable-driven three-degree-of-freedom (DOF) wrist rehabilitation exoskeleton actuated by the distributed active semi-active (DASA) system. Compared with the conventional cable-driven robots, the workspace of this robot is increased greatly by adding the rotating compensation mechanism and by optimizing the distribution of the cable attachment points. In the meanwhile, the efficiency of the cable tension is improved, and the parasitic force (the force acting on the joint along the limb) is reduced. Besides, in order to reduce the effects of compliant elements (e.g., cables or Bowden cables) between the actuators and output, and to improve the force bandwidth, we designed the DASA system composed of one geared DC motor and four magnetorheological (MR) clutches, which has low output inertia. A fast unbinding strategy is presented to ensure safety in abnormal conditions. A passive training algorithm and an assist-as-needed (AAN) algorithm were implemented to control the exoskeleton. Several experiments were conducted on both healthy and impaired subjects to test the performance and effectiveness of the proposed system for rehabilitation. The results show that the system can meet the needs of rehabilitation training for workspace and force-feedback, and provide efficient active and passive training.

## Introduction

Many people suffer from movement disorders and reduced muscle strength, which are resulted in neural diseases (Stroke Center). They have difficulties in performing activities of daily living (ADLs). Studies have shown that rehabilitation training can promote brain damage or redundant nerves to re-learn and restore function (Bayona et al., 2005; Donatelli, 2012; Hatem et al., 2016). In recent years, researchers have developed a variety of different types of robots for post-stroke rehabilitation training. Many related clinical trials based on these robots have been carried out, and the results verified the effectiveness of robot-assisted rehabilitation (Reinkensmeyer et al., 2000; Lum et al., 2002; Kwakkel et al., 2008). The robot is very suitable for repetitive stroke rehabilitation training and has the advantages of high precision.

The flexible movement of the wrist is indispensable for daily life, especially some delicate movements like drinking and eating. The size of the wrist is much smaller than the shoulder and elbow joint. However, it still has a large workspace, including three DOFs of flexion/extension (FE), radial/ulnar deviation (RU), and pronation/supination (PS). In this paper, the forearm PS is considered as one DOF of the wrist. The required range of motion and torque range of ADLs are shown in **Table 3** (Perry et al., 2007; Gupta et al., 2008). To meet the workspace and output force/torque requirements of rehabilitation training in a small space, some robots use a parallel mechanism to achieve weight reduction while maintaining structural rigidity (French et al., 2014; Bian et al., 2017). However, to avoid interference with the movement of the forearm, the workspace of the wrist robot is limited. Some robots use a series spherical mechanism to reduce interference with the arm, which increases the workspace and has sound effects, such as Pehlivan et al. (2014) and Buongiorno et al. (2018). But its structure is relatively complicated. In particular, wrist motion is not just a simple spherical joint motion, but with a certain wrist center shift (Schiele and van der Helm, 2006; Rijnveld and Krebs, 2007). Moreover, some errors are inevitable in the process of wearing the robot. These reasons may lead to misalignment between the center of the human joint and the robot joint during training, resulting in potential physical injury and low recovery training efficiency (Cempini et al., 2013). Existing robots often adapt to changes in the joint center through complex mechanisms, which increases the complexity of the mechanism to a certain extent and reduces stability (Omarkulov et al., 2016; Su et al., 2019). Also, for the rigid robots discussed above, the negative influence of the inertia on the force-feedback performance cannot be ignored, especially in PS.

**Figure 1:**
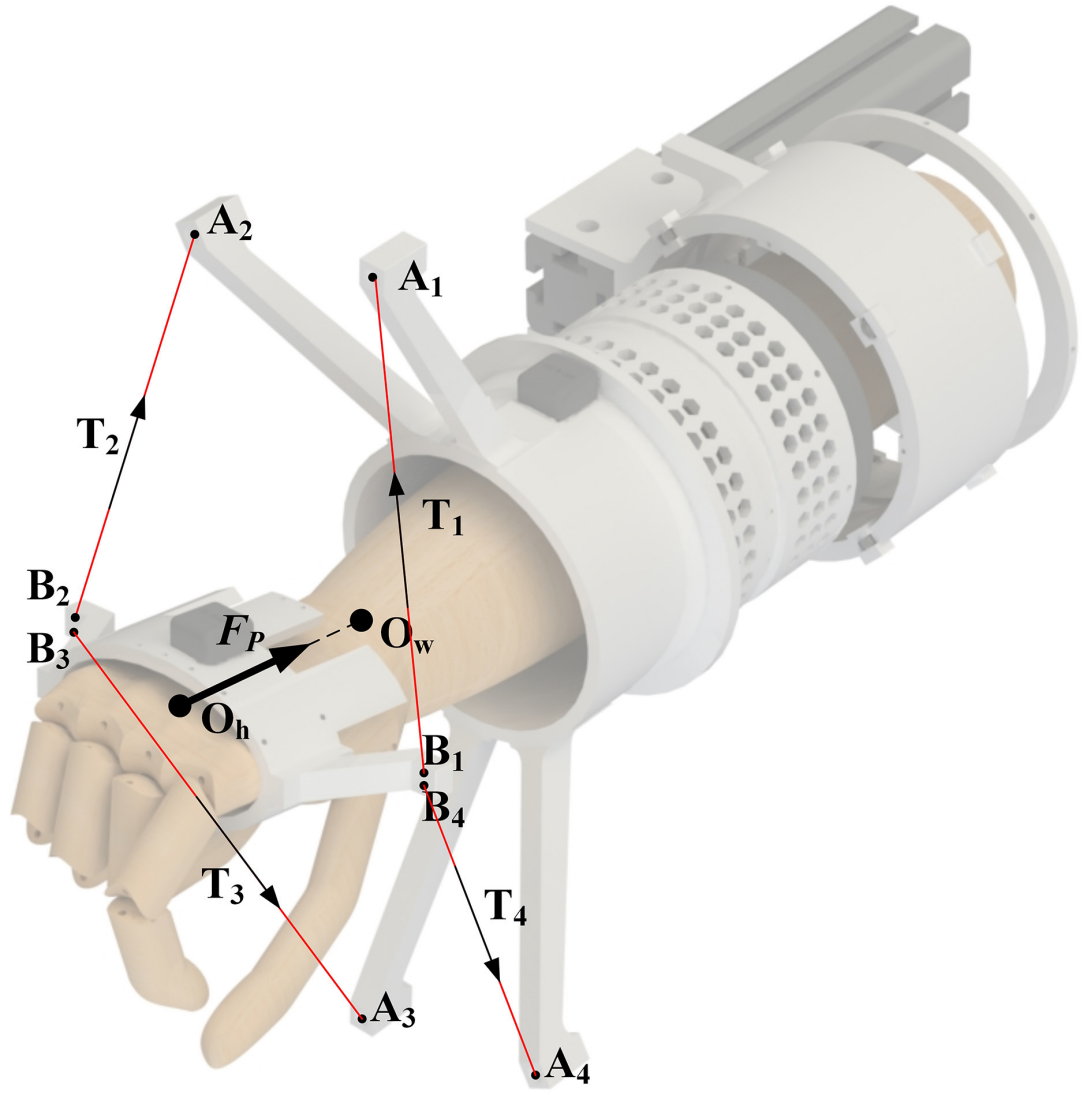
The definition of the parasitic force: *O*_*w*_ is the center of the wrist joint, *O*_*h*_ is the center of the hand, *A*_1−4_ are four cable attachments of the forearm module, *B*_1−4_ are four cable attachments of the hand ring, *T*_1−4_ is the cable tension, and *F*_*p*_ is the parasitic force.

The cable-driven rehabilitation training robot has attracted extensive research due to its lightweight, simple structure, low inertia, high flexibility, and excellent adaptability. It should be noted that the cable-driven robot is divided into cable-transmitted like (Veneman et al., 2005; Alamdari and Krovi, 2015), cable-driven like (Mustafa et al., 2006; Mao and Agrawal, 2012), but only the cable-driven robot was discussed in this paper because the cable-transmitted robots are similar to the rigid exoskeletons, which have a rigid structure to interact with the users. Although many different cable-driven exoskeletons have been proposed (Mustafa et al., 2006; Chen et al., 2015; Cui et al., 2017), their performances are still not good enough to support flexible wrist rehabilitation. Because of the inherent characteristic that the cable only generates tension along its direction, existing cable-driven exoskeletons cannot efficiently match the wrist motion in any rehabilitation training. By analyzing the engineering requirements, we aim to improve the cable-driven design in terms of the following three points:

– Larger workspace. The sufficient joint movement range and DOFs are essential design criteria for the rehabilitation exoskeleton (Riener, 2007). Limited by cable-driven form, the workspace of typical cable-driven design is relatively small so that it cannot match the workspace of the complete ADLs training. Moreover, unlike rigid robots, cable-driven robots will be uncontrollable when it is out of the feasible workspace. This disadvantage causes potential danger, especially for patients with weak motor capacity, so the trajectory out of the feasible workspace must be avoided. The large workspace can ensure that the tension is always controllable.
– Higher cable tension efficiency. The inherent characteristics of the cable may exert a parasitic force on the limb. As shown in Figure 1, the parasitic force is the force generated by the robot along the limb, which is challenging to eliminate in existing cable-driven exoskeletons. The cable tension efficiency is defined as the magnitude of the torque acting on the wrist joint generated by the same tension. The greater the torque, the higher the tension efficiency. At the same time, the smaller parasitic force acting on the joint, the training is more comfortable.
– Higher bandwidth. The previous research has confirmed the importance of the force bandwidth to the rehabilitation robot (Manna and Dubey, 2018). However, because the application of traditional electromechanical actuators introduces high intrinsic inertia, the compliant element (cables or Bowden cables) between the actuators and the output component of the robot lowers the natural frequency of the system, thus limiting its dynamic performance (Viau et al., 2017).

**Figure 2:**
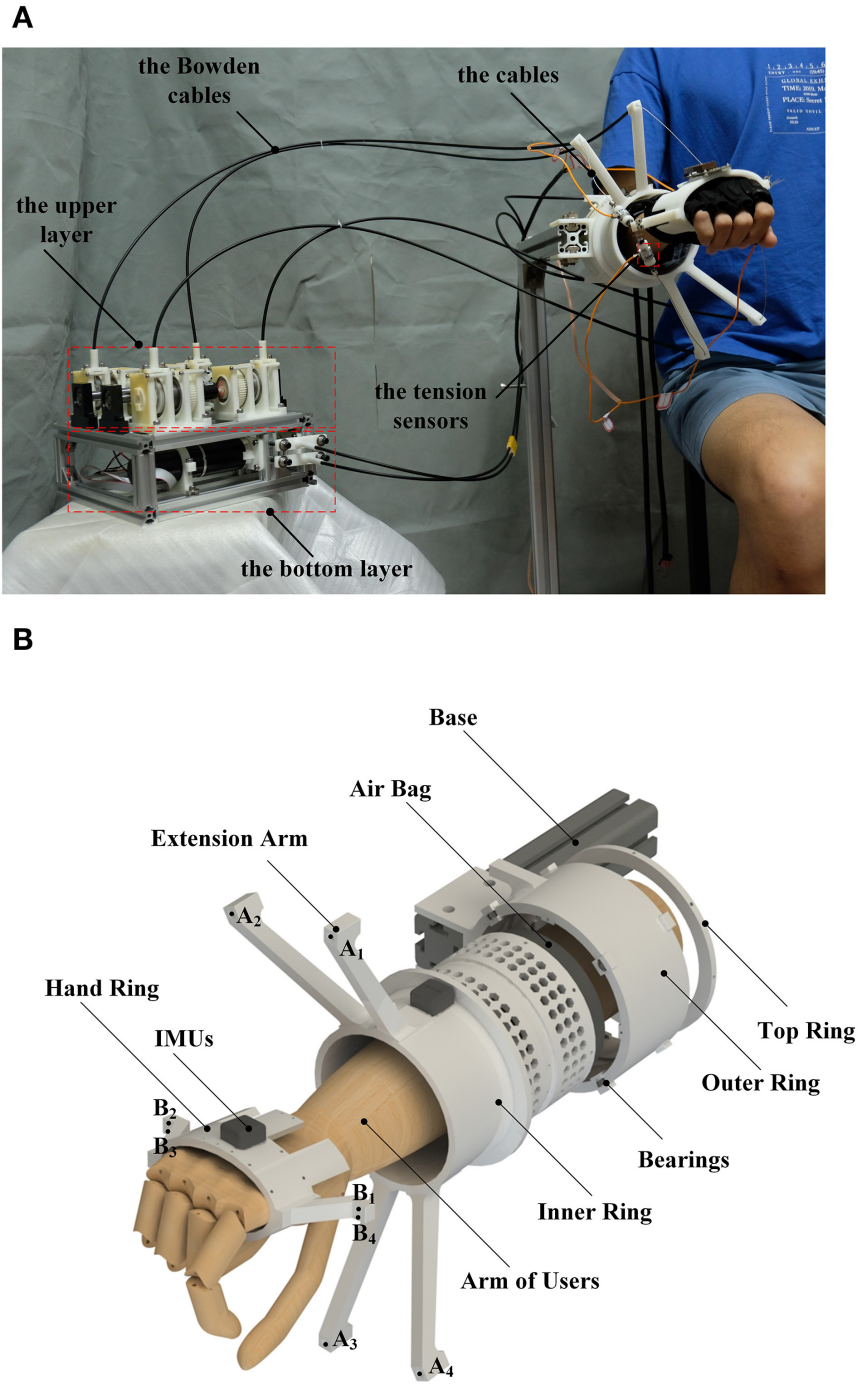
**(A)** close-up of the robot on a healthy subject, **(B)** the exploded view of the robot: *A*_1−4_ are four cable attachments of the forearm module, and *B*_1−4_ are four cable attachments of the hand ring.

Therefore, this paper proposed a cable-driven three-DOF wrist rehabilitation exoskeleton with optimized performance, the SEU-WRE. As shown in Figure 2A, the cable-driven system is adopted, and the overall structure is straightforward. The main structure is made of nylon material by 3D printing, and the weight except the driven system is 350 g (the grip portion is only 50 g). The inertia is pretty low in all three DOFs. To improve the first two disadvantages, the rotating compensation mechanism on the forearm module is designed, and the distribution of the cable attachments can be adaptively changed, optimizing the workspace of the cable-driven robot and the tension efficiency of the cable. The workspace meets the training requirements of ADLs completely, and the cable can generate sufficient torque output during training within limited tension. At the same time, the parasitic force is reduced.

Routing the cables via the Bowden cable reduces the structural complexity and size worn by the users. For optimizing the force performance and reducing the influence of the elastic elements of the cable-drive system, the DASA system based on MRs is adopted. Viau et al. (2017) applied this kind of drive system on a cable-driven manipulator, and it exhibits excellent force-feedback performance. In this paper, the structure of this drive system is optimized for the rehabilitation exoskeleton to improve the force bandwidth and the tension control.

Regarding safety, the advantage of rigid robots is that their joints can be mechanically limited, but they usually have to be tightly fixed to the limb, which cannot unbind quickly in abnormal conditions. For SEU-WRE, two safety modes have been set up to improve safety. One is used for the protection during the training process when it exceeds the pre-set movement range, or the cable tension is abnormal. The other is used to achieve fast unbinding, based on the DASA drive system with high back-drivability and the nature of the cable-driven system, which is difficult for rigid robots. The performance comparison of several typical robots is shown in Table 1.

**Table 1:** The performance comparison of several typical exoskeleton.

	**Rigid parallel**	**Rigid serial**	**Existing cable-driven**	**SEU-WRE**
Typical Example	MAHI EXO-II (French et al., 2014)	RiceWrist-S (Pehlivan et al., 2014)	CDWRR (Chen et al., 2015)	/
Workspace	Low	High	Low	High
Torque	Medium	High	Low	Medium
Inertia	Medium	Low	High	High
Joint alignment	Low	Medium	High	High
Force-feedback performance	Medium	Medium	Medium	High
Safety	Medium	Medium	Low	Medium

*Rough comparison and classification are shown in the table. “Low” indicates poor performance, and “High” indicates relatively good performance. The force-feedback performance is determined by the inertia and drive performance (back-drivability and system friction, etc.). The safety analysis is presented in section Introduction and Safety Modes*.

Section System Concept introduces the structural design of SEU-WRE; section System Analysis and Optimization carries out the robot dynamics analysis, workspace optimization and tension efficiency analysis; section Control Algorithm presented the passive and active training algorithms to test the performance and effectiveness of the proposed system for rehabilitation; section Experiments and Analysis carries out the related experiments including one healthy and two impaired subjects, and analyzes the experimental results; finally, section Conclusion summarizes and discusses this paper.

## System Concept

### Mechanical Design of the Robot

The cable-driven robot is typical to use at least *n*+*1* up to *2n* cables to drive *n* DOFs, or to act as a cable by external forces such as gravity (Mustafa and Agrawal, 2012). Since the cable can only provide a pulling force in the direction of the cable, even if the redundant cables are used, the workspace of the robot is usually quite limited. In particular, when the cable pulls the limb to move, the relative position of each cable attachment changes, and the cable configuration changes, affecting the tension efficiency, further limiting its workspace and increasing the parasitic force. For instance, in FE and RU DOFs of the wrist joint, the cable attachment distribution can be optimized to satisfy the ADLs space as much as possible and provide enough torque applied to the wrist for rehabilitation. But no matter what kind of cable configuration, without prejudice to the compactness requirements of the overall structure, it is challenging to meet the motion space requirements for the forearm PS. In particular, when the cable-driven wrist robot drives the wrist to rotate in PS, the configuration of the cables changes, and the tension efficiency of the cable in this DOF gradually decreases until a singularity occurs and no torque is supplied. Moreover, for the PS, interference between the cables and between the cable and the limb is not easy to avoid, so how to deal with the PS movement is an essential problem for the three-DOF joint exoskeleton. Based on this consideration, this paper proposed a dynamic change strategy of the cable configuration, that is, by adding a rotating compensation mechanism, the inner and outer rings of the forearm module can be relatively rotated, and the fixed cable attachments can be dynamically adjusted according to the current wrist posture. Thereby the cable configuration can be changed in real-time, increasing the workspace and improving the cable tension efficiency.

As shown in Figure 2B, it is an exploded view of this robot without cables and Bowden cables. The outer ring is fixed with a bracket or any other upper limb rehabilitation robot, which can be combined with the wrist robot. And the inner ring can be driven by a cable wound on it to rotate relative to the outer ring. The inner side of the outer ring is provided with a plurality of miniature bearings for keeping the rotation of the inner ring stable. When the inner and outer rings rotate relative to each other, the cable attachments on the four extension arms of the inner ring also rotate at the same time to complete dynamic adaptation. The inner ring rotation is controlled by position-based mode and always follows the Z-axis rotation of the hand ring, so the cables configuration never changes due to the forearm PS movement. The four cables are respectively connected to the four cable attachments *B*_1−4_ on the hand ring through *A*_1−4_ on the four extension arms, generating different tensions, and the hand ring is controlled to drive the limb movement or provide the force-feedback. The relationship between the output of the MR clutch and the input current can be fitted. However, there is still the hysteresis of the MR and many other disturbances (e.g., the unmodeled friction of the Bowden cables) in the system that cannot be accurately predicted, so a tension sensor is added to each cable to establish the tension close-loop. Inside the inner ring, there is a non-slip silicone inside the airbag to assist the forearm to be located on the central axis of the inner ring as much as possible, and to provide certain axial friction during movement to reduce the axial displacement of the arm due to parasitic force. At the same time, the hand posture and the rotation angle of the inner rings are collected by two inertial measurement units (IMU). The robot forearm module is connected to the hand ring through four cables, and there is no rigid connecting device. For the rotating compensation mechanism, although the connection between the inner ring and the outer ring is rigid, and the position control is adopted, the PS rotation is controlled directly by four cables. So three DOFs of the wrist are still driven by the flexible cable. This is crucial because the flexibility of the cable, the inertia of PS and the human-robot interaction through the tension control play an important role in the safety and comfort of rehabilitation training. Moreover, the force-feedback bandwidth generated by this motor is far from satisfactory. Only by directly applying the force to the wrist through the cable can the better dynamic performance be ensured. The motor-driven rotating compensation mechanism is only used to ensure sufficient speed tracking, adapt to the hand posture so that the cable configuration can be adjusted in real-time to meet the requirement of cable-driven strategy.

The structure is simple and easy to wear. Before training, if the patient's ability is poor, the forearm module can be taken off the base and put on the arm, and the inner diameter is large enough to pass through easily. The hand only needs to wear the hand ring, and the tension cables will firmly fix it on the hand.

### MR-DASA Drive System

**Figure 3:**
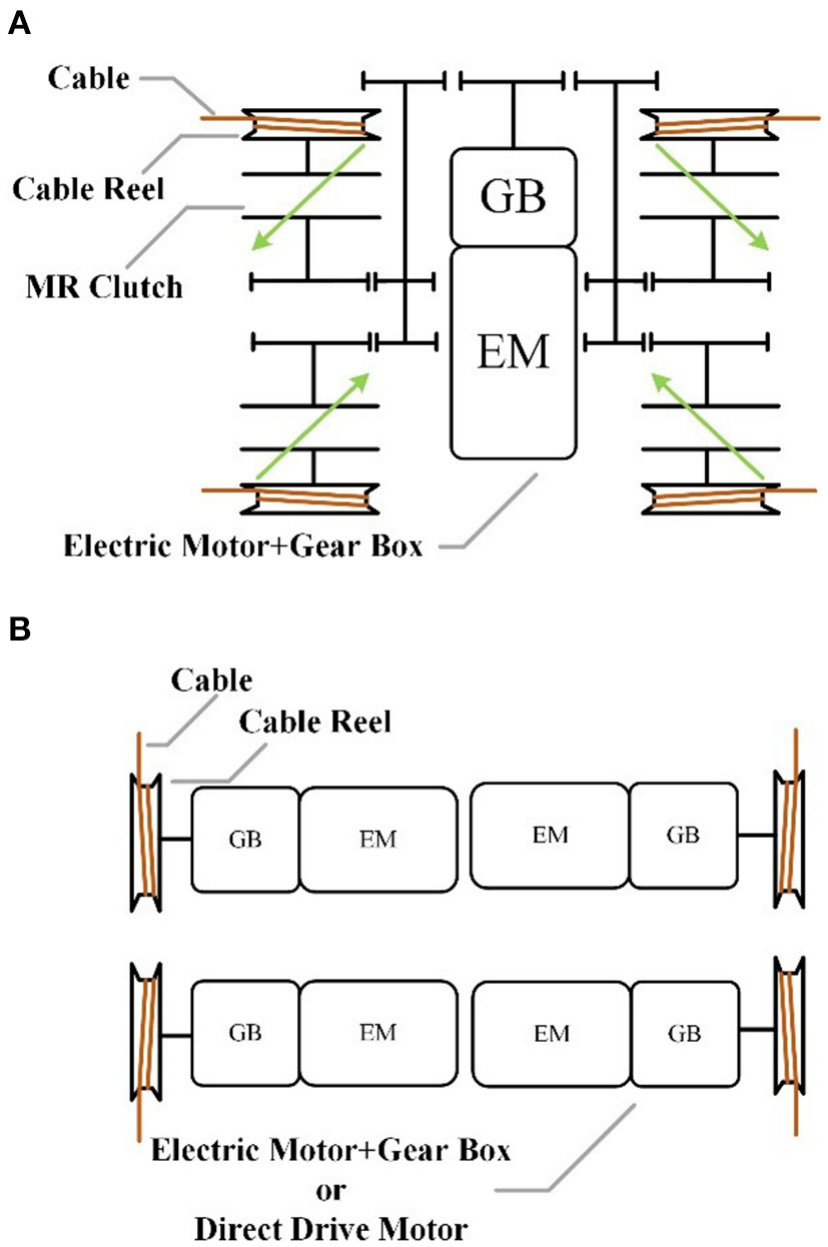
The schematic of two different drive systems: **(A)** the DASA system based on MRs, **(B)** the conventional electric motor drive system.

As mentioned in section Introduction, cable-driven robots have many advantages. However, much like SEA (series elastic actuator), the compliant elements between the actuators having high inherent inertia and the output lowers the natural frequency of the system, the dynamic performance is limited. Compared to the conventional motor, the use of an MR clutch can weaken this effect, since its low output inertia. Therefore, the MR is used for this cable-driven robot, which can improve the force-feedback performance and is very suitable for human-robot interaction, including the rehabilitation robot (Viau et al., 2017). MR is a passive clutch that only provides damping and cannot provide active output. Therefore, the DASA system is utilized to actuate the cable-driven robot, and the geared DC motor with high power-density is used as the power source of the DASA system, which is shown in Figure 3A. The conventional electric drive system is shown in Figure 3B, the direct-drive or geared motor are usually used as the tension generators, which has poor dynamic performance due to the high output inertia.

**Figure 4:**
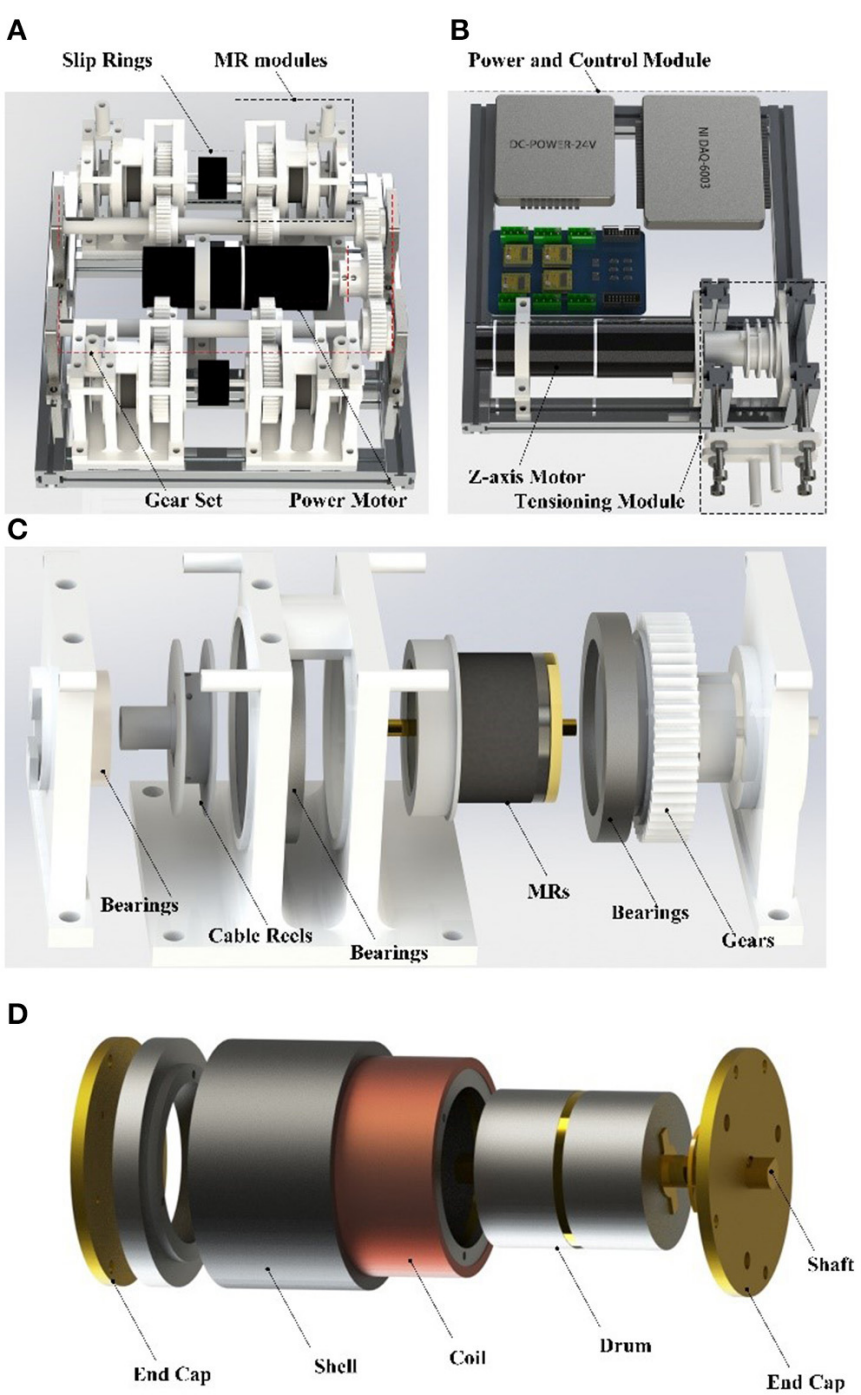
The power box: **(A)** the upper layer, **(B)** the bottom layer, **(C)** the exploded view of the MR-Cable unit, **(D)** the MR clutch assembly.

As shown in Figure 4, the lower layer includes the actuator system for the rotating compensation mechanism, composed of the Z-axis motor (MAXON RE40) and the tensioning module. After transmission through the cables, the maximum speed of the rotating compensation mechanism is 80 rpm. The other side of the lower layer is composed of the main power module (24V/60W), data acquisition card (NI6060), and switching circuit. The control program of this system runs on the PC. The upper layer is the DASA system, including the power motor and four MR-Cable units, which are placed in parallel. When the system works, the power source motor keeps rotating to drive MRs to rotate in slippage. The MR distributes the mechanical power provided by the motor and generates the required torque to the cable reel. The MR clutch pulls the cable through the cable reel, and the cables drive the hand ring and the outer ring through the Bowden cable. When in slippage, the MR clutch decouples the dynamic behavior of the power source motor from the output, resulting in actuators with high force resolution as actuator has low reflected inertia and negligible non-linear effects. The MR clutch is made in our laboratory. The performance of the MR clutch and the DC motor is shown in Table 2.

**Table 2:** Performance of the drive system.

**Parameter**	**Value**
MR maximal torque	1.2 nm
MR maximal current	1 A
MR outer diameter	42 mm
MR Width	38 mm
Power motor max speed	700 rpm
Power motor max torque	4 Nm
Z-axis motor max speed	180 rpm
Z-axis motor max power	48 W

## System Analysis and Optimization

### System Dynamics Analysis

The dynamic equations of motion for this wrist exoskeleton are derived using the Lagrangian method. The generalized coordinates *q*_1_, *q*_2_, and *q*_3_ represent wrist FE, RU, and PS, respectively. The dynamic modeling for this three-DOF robot is presented as follows:

(1) $$\begin{array}{l} D\left(q\right)\ddot{q}+C\left(q,\dot{q}\right)\dot{q}+g\left(q\right)={J\left(q\right)}^{T}T\left(t\right) \end{array}$$

where ***D*** denotes the inertia matrix, ***C*** denotes Coriolis and centrifugal term, and ***G*** denotes the gravity term of both device and the human limb, ***q*** represents the joint angle, and ***J**(**q**)* is the Jacobian matrix relating cable tensions to joint moments, and ***T**(**t**)* is the vector of cable tensions. Due to the characteristic of cables, the tension must be kept for system control. The cable tension planner has been proposed in many related researches. Usually, the tension planner can be expressed as:

(2) $$\begin{array}{l} AT=\tau \end{array}$$

where ***A*** = ***J(q)***^*T*^ in (1), **τ** is the torque that is required at the joints to drive the arm. Because the number of cables is more than the number of DOFs, the solution of (2) can be written as:

(3) $$T=\bar{T}+N(A)m$$

where $\bar{T}=\left[t_{1};t_{2};t_{3};t_{4}\right]$ is the minimum norm solution of (2) which is given by:

(4) $$\begin{array}{l} \bar{T}=A^{T}{\left(AA^{T}\right)}^{-1}\tau \end{array}$$

***N*****(*A*)** = [*n*_1_; *n*_2_; *n*_3_; *n*_4_] is a null space matrix of ***A*** and *m* is an arbitrary value, assuming ***A*** is full rank. Considering constraints of the tension, the planner can be expressed as:

(5) $$\left[\begin{array}{c} N(A)\\ -N(A) \end{array}\right]\;m\geq \left[\begin{array}{c} T_{\min}-\bar{T}\\ -T_{\max}+\bar{T} \end{array}\right]$$

Finally, the optimal solution of cable tensions can be found as the following:

(6) $$\begin{array}{l} \min\overset{4}{\underset{i=1}{\sum }}\left(t_{i}+n_{i}m\right) \end{array}$$

$$\begin{array}{l} s.t.{\;T}_{\min}\leq \bar{T}+N\left(A\right)m\leq T_{\max} \end{array}$$

***T***_***max***_ is the upper bound of tension, which is set as *42N* in this paper, and ***T***_***min***_ is the lower bound, which is *1.5N*.

To calculate the cable tension efficiency and solve the cable tension in unknown conditions for robot control, maximum torque in assigned direction within the limitation of cable tension can be given by (6), that is, in the cable tension range, if there is no solution for the given torque, the maximum torque that can be generated in the same direction is generated.

(7) $$\begin{array}{l} \left[\begin{array}{c} N\left(A\right)\\ -N\left(A\right) \end{array}\right]\;m_{2}\geq \left[\begin{array}{c} T_{\min}\\ -T_{\max} \end{array}\right]+\left[\begin{array}{c} -\bar{T}\\ \bar{T} \end{array}\right]\;m_{1} \end{array}$$

$$\begin{array}{l} \max\overset{4}{\underset{i=1}{\sum }}t_{i}\\ s.t.{\;T}_{\min}\leq \bar{T}m_{1}+N\left(A\right)m_{2}\leq T_{\max} \end{array}$$

### Workspace Optimization and Analysis

**Figure 5:**
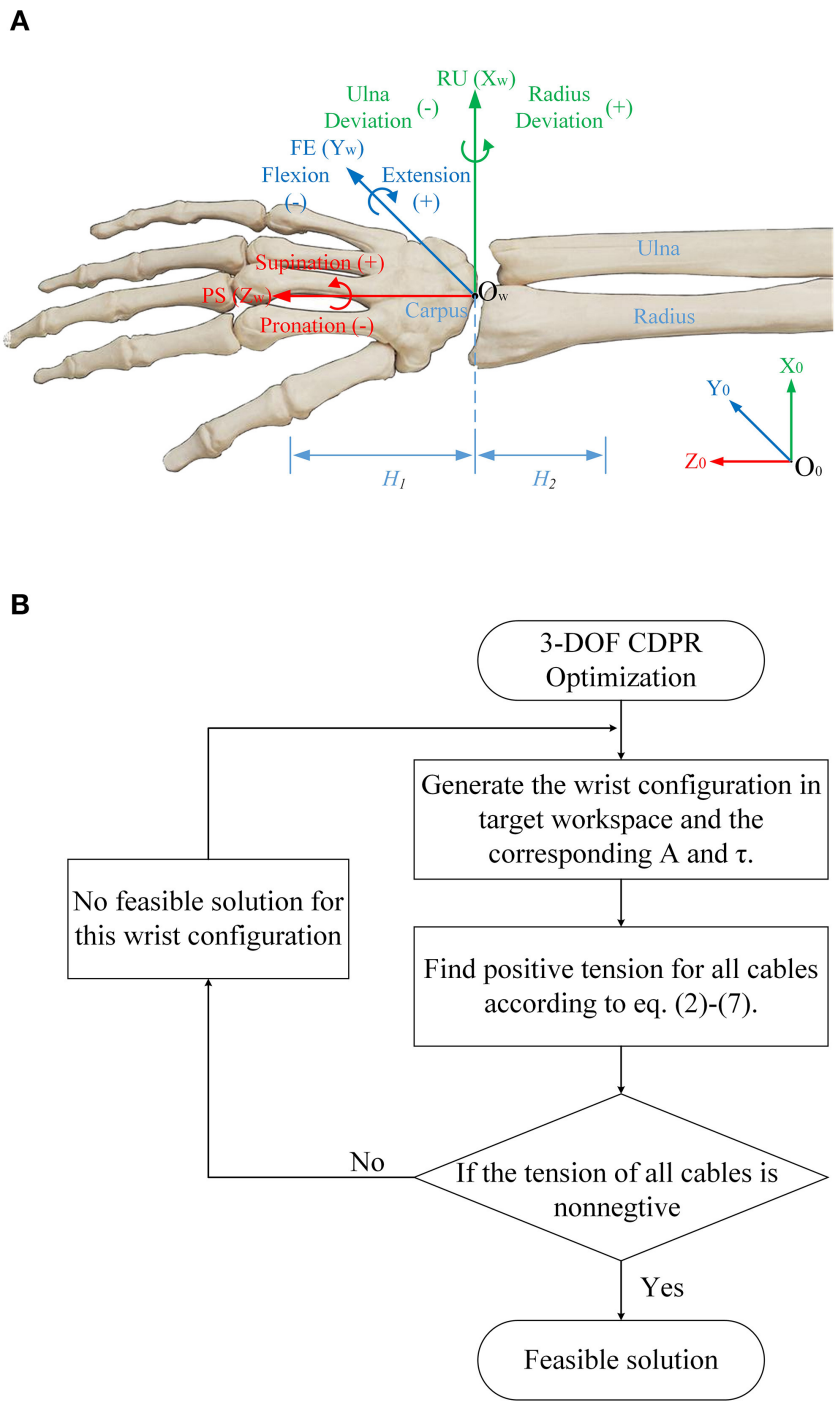
**(A)** anatomy of the wrist joint and the coordinate frames, **(B)** the optimization process of the workspace.

Research on the cable-driven exoskeleton robot shows that the distribution of the cable attachments, the diameter of each component, and the angle of inclination of the fixed brackets have a significant impact on the workspace (Mustafa et al., 2006; Mustafa and Agrawal, 2012; Shao et al., 2014). Regarding the optimization of the distribution of the cable attachments, many researchers have done related research, including particle swarm algorithms (Bryson et al., 2016), which are used to explore the optimal solution. Cable-driven robot workspace optimization is a complex problem with many variables. Therefore, to simplify the optimization process, some parameter ranges are usually constrained. Figure 5A shows the anatomy of the wrist joint and the coordinate frames. Considering the actual situation, the following constraints are imposed:

1. When optimizing the workspace, we neglect PS and the wrist joint is regarded as a spherical joint. The range of human joint is show in Table 3. Considering the approximate symmetry of its motion, we assume that the forearm cable attachment plane is perpendicular to the forearm;
2. The diameter of each component of the wrist rehabilitation training robot also has a significant influence on the optimization, mainly *R*_1_ (the radius of the hand ring) and *R*_2_ (the radius of the inner ring extension arm). Through the preliminary the study of Shao et al. (2014), the excessive *R*_1_*/R*_2_ value, will lead to lower tension efficiency, that is, increased parasitic force. In order to ensure that the cable does not interfere with the hand during the movement, the *R*_1_ value is 38 mm, to avoid the interference between the trunk and the inner ring, to ensure compact, the *R*_2_ value is 75 mm, and the cable attachments are distributed on the ring;
3. The rotating compensation mechanism is adopted, so that the distribution of the cable attachments can be dynamically changed. Hence, it is not necessary to set the PS movement range as ±90° in the calculation. The motor that drives the rotating compensation mechanism can quickly respond to the movement of the wrist PS and compensate the rotating angle. So the PS range is set in ±20° as an optimization condition to cope with the motor response delay;
4. During the optimization process, point *A*_1−4_ is calculated by *2.5*° step along the radius of *75 mm*, and point *B*_1−4_ is calculated by *2.5*° step along the radius of *38 mm*. Each point is constrained to a certain range to avoid repeated combinations;
5. Regarding the wearing error and individual differences, the *H*_2_ length (the wrist joint center to the forearm cable attachment plane) and the *H*_1_ length (the wrist joint center to the hand ring cable attachment plane) are set as *1* ± *1 cm* and *14* ± *2 cm*, respectively. It allows the error within the range to be generated while training and still can ensure that the workspace is effective.

**Table 3:**
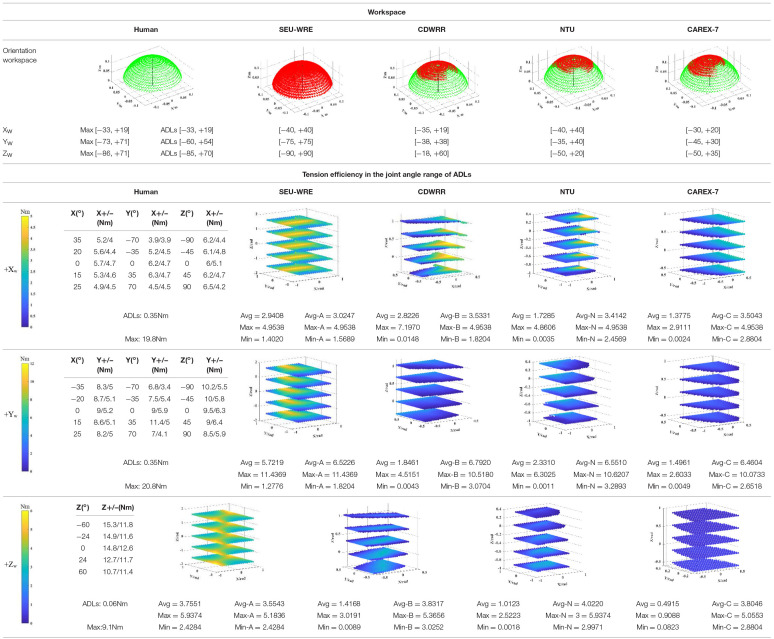
The workspace and tension efficiency comparison of four cable-driven robot.

*X-A/B/N/C represents the torque generated by SEU-WRE in ADLs/CDWRR/NTU/CAREX workspace, and X represents Avg/Max/Min. The human data refer to Gupta et al. (2008), Perry et al. (2007), and La Delfa and Potvin (2017)*.

The optimization process is shown in Figure 5B. Finally, *128* sets of feasible solutions can be obtained after optimization, and the final cable attachment distribution will be selected according to the cable tension efficiency introduced in the next section.

As shown in Table 3, the top part is a comparison of the workspace of four cable-driven robots, which are SEU-WRE, CDWRR (Chen et al., 2015), NTU (Mustafa et al., 2006), and CAREX-7 (Cui et al., 2017). The hand is approximately equivalent to a *14-cm-long* rod. One end is fixed at the center of the wrist joint, and the three-DOF rotation axis coincides with the rotation axis of the wrist joint. When calculating, the spatial position of the other end of the rod corresponding to the feasible joint angle is printed as a visual display of the feasible workspace. The red point is the feasible workspace for each robot, and the green point is the human joint workspace for the corresponding. It can be seen that SEU-WRE can completely cover the human wrist joint workspace. In contrast, the other three robots have a great gap in the workspace and the human joint space, and there is also a certain gap with the ADLs space. Insufficient workspace makes it necessary to pre-calculate the trajectory during the rehabilitation training. Otherwise, it will cause unpredictable errors and danger in the movement beyond the workspace.

### Tension Efficiency Analysis

In this paper, the cable tension efficiency is defined as the magnitude of the torque acting on the wrist generated by the same tension conditions. The greater the torque, the higher the tension efficiency. At the same time, the smaller the parasitic force acting on the joint, the higher the comfort of training. The workspace priority strategy is adopted. Under the premise of satisfying the workspace of the human joint, the tension efficiency of each cable attachment distribution is analyzed, and the set with the highest tension efficiency is selected as the final solution.

As shown in the bottom part of Table 3, the tension efficiency analysis is performed for each robot. CDWRR, NTU, and SEU-WRE are independent wrist rehabilitation robots or upper-limb robots with detachable design. The wrist joints can be analyzed independently. But, the cables are routed from the proximal cuffs to the distal cuffs in CAREX. It is difficult to analyze directly, so the wrist structure of CAREX is equivalent to an independent structure, and the tension efficiency analysis is performed.

Based on the maximum torque in the assigned direction algorithm, as expressed in (7), the maximum joint torque that can be generated in the workspace is analyzed. Table 3 lists the torque in the positive direction of three DOFs to compare cable tension efficiency. For the column of human joint torque, the research shows that the joint torque is different under different joint angles (La Delfa and Potvin, 2017; Su et al., 2019). Therefore, the wrist joint torque of several discrete joint angles is selected as the human joint value. Among them, the torque of FE and RU is affected by the angle of three DOFs, and the PS joint moment is only affected by the PS angle. Moreover, studies have shown that there is a big difference between the daily joint torque and the maximum joint torque of the human body. This is a concern when developing the force-feedback function of the wrist joint robot (mainly for healthy people), that is, the force-feedback needs to have a broad range of stiffness variation. In SEU-WRE, CDWRR, NTU, and CAREX-7 columns, the maximum positive torque of each DOF is shown when the cable tension is limited to a specific range. To ensure the consistency of the comparison, the minimum tension of each robot is limited at *5 N*. But the maximum depends on the number of cables, that is, the maximum cable tension of the 4-cable-driven robots is *45 N*, and the 6-cable-driven robots is *30 N*. Table 3 shows the maximum, minimum, and average joint torque output of the four robots in their workspace, and the joint torque output of the SEU-WRE in the human ADLs, CDWRR, NTU, CAREX workspace to ensure the comprehensiveness of the comparison. In the visual processing of the joint torque, for the clear display, five Z-axis joint angle values are selected, and *100* X/Y-axis joint angle values are chosen in the range. But in the actual numerical calculation, *500* values of each joint are chosen to ensure the accuracy of the calculation. As can be seen from Table 3, SEU-WRE has a significant advantage in the generation of any joint torque, that is, it has exceptionally high cable tension efficiency, which means that the parasitic force generated during the rehabilitation process is smaller and the training is more comfortable and safe. The maximum torque generated is still different from the maximum output of the human joint torque, because the cable tension necessarily needs to be limited based on safety and comfort.

It is not difficult to find that SEU-WRE has significant advantages in both aspects. It is due to that the rotating compensation mechanism is used, dynamically changing the cable attachments distribution and greatly increasing the workspace and tension efficiency. This is very important for rehabilitation training.

## Control Algorithm

### MR-Cable Tension Control Algorithm

The system uses the Bowden cable as a transmission to reduce overly complex cable routing. Many studies on the Bowden cable transmission have shown that the Bowden cable inner sleeve and the passing cable can cause friction, including coulomb friction, viscous friction stiction, and stick-slip (Letier et al., 2006; Palli and Melchiorri, 2006). In order to reduce the influence of friction, according to the previous research, this paper chooses a 1^*^7 multi-strand steel cable with a diameter of *1 mm*. Both the cable shell and the Bowden cable inner sleeve are made of PTFE material to minimize the viscous friction. The Bowden cable inner sleeve is selected from the longitudinal construction of flat-band steel to reduce the effect of its elasticity on the system. The diameter of the Bowden cable is *5 mm*.

**Figure 6:**
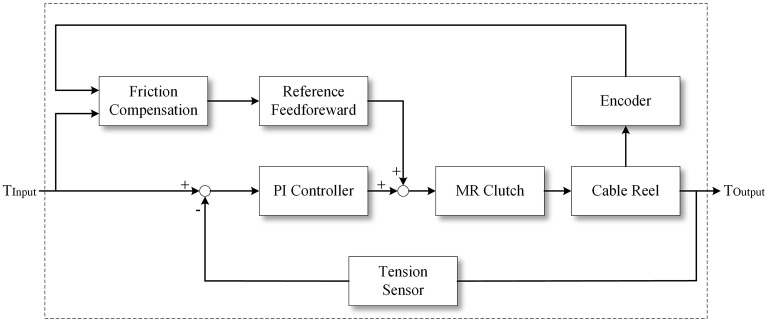
The MR-Cable tension control block diagram.

Even if the above material is used, the stiction can be eliminated, but Coulomb friction still exists. According to the model proposed in (Palli and Melchiorri, 2006), the feedforward compensation of the friction is achieved. Although the current-to-torque relationship of the MR clutches can be fitted by the model, when the cable tension control is performed, the open-loop control is not directly adopted, but the cable tension sensor is used. A closed-loop tension control model was established to compensate for the effects of the unmodeled friction and the MR hysteresis. The control block diagram is shown in Figure 6, in which the reel velocity is used to distinguish the direction of the friction force. Through experimental measurement, even if the dynamic adaptive structure rotates between ±90°, the Bowden cable friction efficiency does not change significantly, so the friction coefficient μ is set as a constant.

### Passive and Active Training Algorithm

The passive training can help patients to move their weak limb in the desired trajectory, which incorporates the stretching of muscles and connective tissues to regain their movement ability by provoking the motor plasticity (Alamdari and Krovi, 2015). The existing cable-driven exoskeleton robots do not mention related training methods. In this paper, the projected PID control algorithm proposed in the literature (Viau et al., 2015) is used to design a mirror training algorithm that can be applied in the three-DOF rehabilitation training robot. In order to perform the force control with friction compensation and enhance safety in rehabilitation training, the open-loop tension control in this algorithm is replaced with the closed-loop control described in section MR-Cable Tension Control Algorithm. Although the force control bandwidth will be reduced, it fully meets the needs of rehabilitation training. The experiment shown in section MR Performance Experiment confirmed this.

The active training is an important part of the rehabilitation training process, including resistive training and assistive training (Proietti et al., 2016). Through the set training strategy, the patient is assisted by the robot to complete the training and strengthen the motor function. The AAN algorithm is used on this robot to verify its active training function:

(8) $$\begin{array}{l} r=\dot{q}-{\dot{q}}^{d}+\Lambda \left(q-q^{d}\right) \end{array}$$

where **Λ** is a positive-definite matrix that determines the weight of position errors relative to velocity errors. Because the weight of the hand ring is only *50 g*, the inertia and Coriolis terms are ignored. The controller can then be written:

(9) $$\begin{array}{l} \tau_{r}=g\left(q\right)-K_{D}r \end{array}$$

where ***K***_***D***_ is a positive-definite gain matrix. The controller functions as a PD controller, where ***K***_***D***_ and ***K***_***D***_**Λ** serve as the derivative and proportional gains, respectively.

In order to provide appropriate challenges to the subjects and strengthen the rehabilitation training, ***K***_***D***_ is updated according to ***r***, which represents the patient's performance.

(10) $$\begin{array}{l} K_{D,i+1}=\left(1+\alpha_{i}\right)K_{D,i} \end{array}$$

(11) $$\begin{array}{l} \alpha_{i+1}=\alpha_{norm}\frac{\bar{r_{i}}-r^{*}}{r^{*}} \end{array}$$

α_*norm*_ is a constant nominal change rate, $\bar{r_{i}}$ is the current task's error, and r^*^ is the maximum allowable average trajectory error. If $\bar{r_{i}}$ is bigger than r^*^, the algorithm dictates that the subject cannot provide enough error performance, and hence, the feedback gain increases for the next task.

### Safety Modes

**Figure 7:**
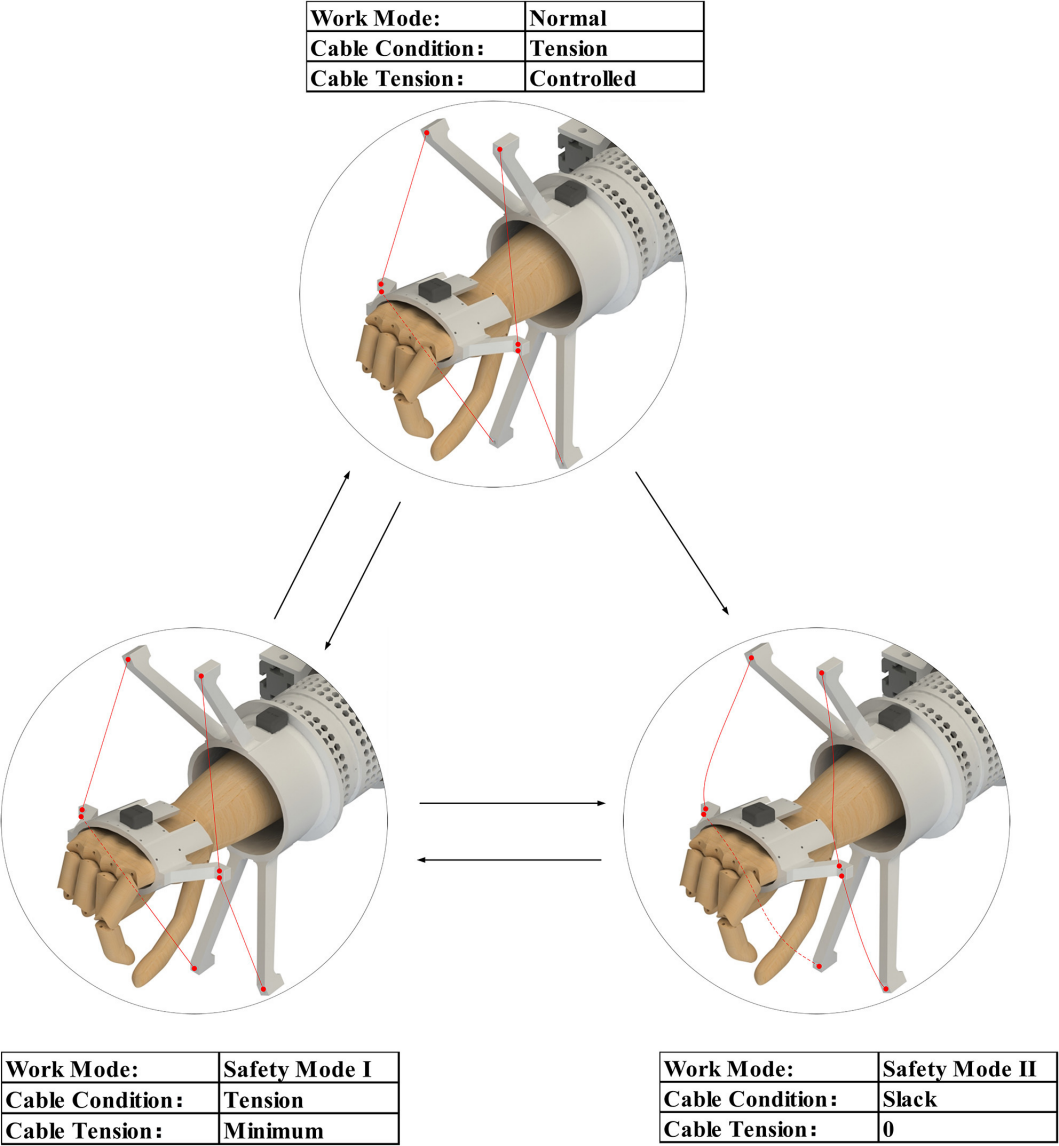
Three work modes for safety.

Safety is an essential requirement in rehabilitation training. As shown in Figure 7, there are three work modes of SEU-WRE to avoid injury when abnormal conditions occur:

1. Normal Work Mode: The tension of each cable can be controlled, and the required torque is provided according to the rehabilitation training algorithm.
2. Safe Mode I: When the patient exceeds the set training area or the interaction force is abnormal, the MRs of the DASA system can be powered off by software or hardware switches, and the rotation speed of the power motor is reduced. At this time, although the power motor continues to rotate, because the MRs have no current input, the tension is the minimal value which is generated by the inherent damping of the MRs. The measured minimum tension is about 1.2 N. When the condition becomes normal, the robot can return to Normal Work Mode directly.
3. Safety Mode II: When an emergency situation occurs, and the training needs to be stopped, the whole DASA system can be powered off through the emergency switch. At this time, the power motor stops rotating, the cable will not be tensioned any more, and can be pulled arbitrarily. At this time, the patient does not interact with the robot, and the hand ring can also be quickly taken off, avoiding the potential damage caused by the robot system friction and inertia.

Safety Mode II is similar to the wearing state before starting training, and it can be switched to Safety Mode I, then to Normal Work Mode. Both Safe Mode I and Normal Work Mode can be directly switched to Safe Mode II. These safety work modes, especially Safety Mode II are difficult to be achieved in the rigid robots, even the OpenWrist, which is convenient to wear (Pezent et al., 2017).

## Experiments and Analysis

### MR Performance Experiment

**Figure 8:**
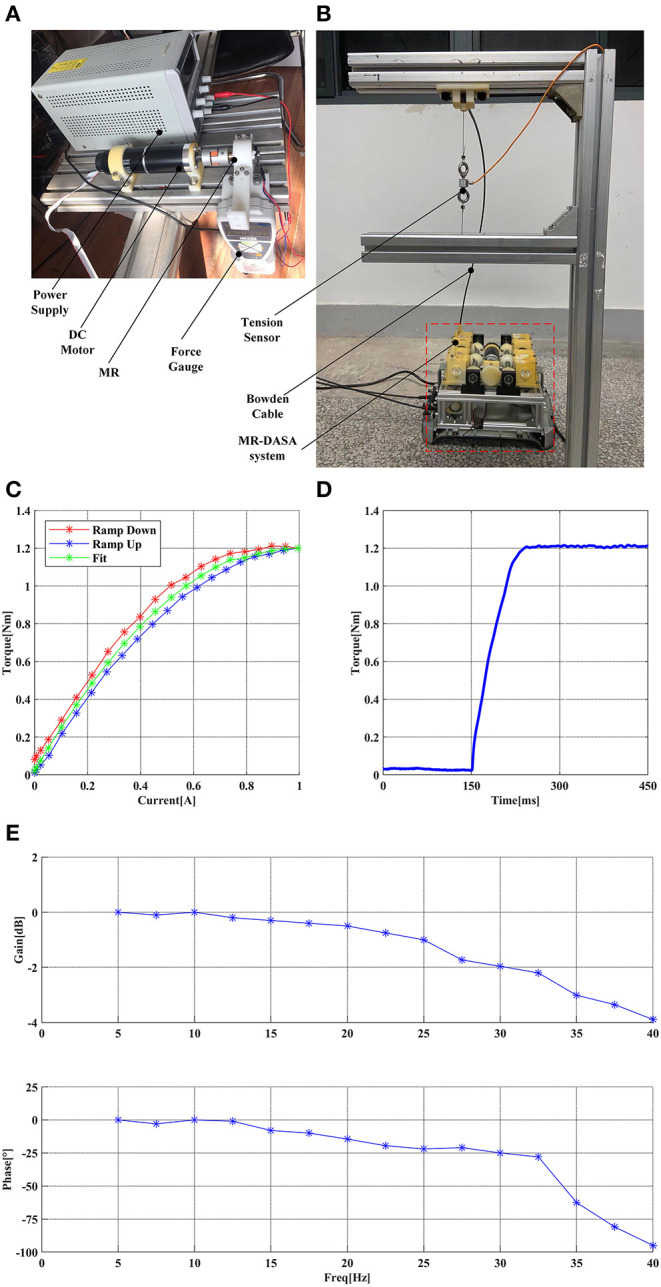
**(A)** the MR performance test platform, **(B)** the MR-Cable system performance test platform, **(C)** the MR current-torque curve, **(D)** the MR step response curve, **(E)** the MR-Cable closed-loop force control Bode diagram.

To demonstrate the performance of the MR clutches and MR-Cable system, the following sets of experiments were carried out. The MR performance test platform is shown in Figure 8A. The motor drives the MR clutch to rotate by the coupling, and the force gauge at the end of the linkage measures and records the output of MR with a given current or step signal input. The MR-Cable system test platform is presented in Figure 8B, the end of one cable is connected to the fixed base and the tension is measured and recorded by the tension sensor.

Figure 8C shows the MR current-torque curve. It can be seen that there is a certain hysteresis between the ramp up and down. In this paper, a second-order polynomial function was used to fit the current-to-torque relationship, and only 50% of its maximum output was used to provide sufficient tension in the DASA system. Figure 8D shows the MR step response curve. It can be seen that the MR clutch has a high step response speed. Combined with the low output inertia of the MR clutch, the MR-Cable system has a high dynamic performance.

As shown in Figure 8E, for the MR-Cable closed-loop force control Bode diagram, the input was a sinusoidal signal with amplitude *30 N*, the frequency growth step is *2.5 Hz*, the gain and phase of each point were recorded. To explore the effect of different bending conditions on the force-feedback bandwidth, the robot system was placed in the normal work state, and the *Cable-1 (A*_1_*-B*_1_*)* was selected as the test sample (the remaining three cables have the same characteristics). The Z-axis was kept at 0°. The bandwidth of this system is about *35 Hz* at −*3 dB* and is fully superior to the human force bandwidth that is between *5* and *10 Hz* (Shimoga, 1992).

### Passive Training Experiment

**Figure 9:**
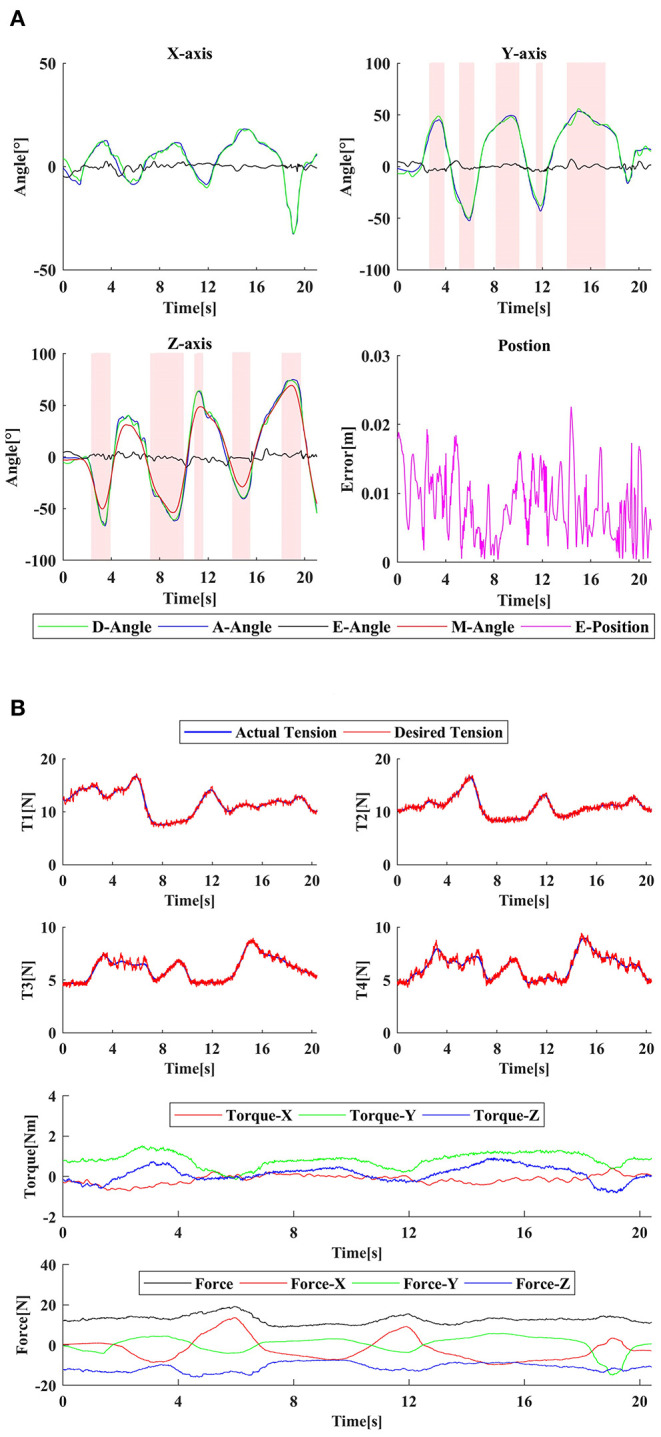
The passive training experiment: **(A)** the tracking error, **(B)** the tension, parasitic force, and actual assistive torque curve. D/A/E/M-Angle represent the desired angle, the actual angle, the tracking error of the angle, and the angle of the Z-axis, respectively. E-Position means the tracking error of the position. The red area in **(A)** is the infeasible area of CDWRR.

Because the patient has limited ability in speed and range of movement, in order to fully verify the passive training functions, including tracking speed, accuracy, workspace, and parasitic force, one healthy subject S1 (26 years old, male) was selected to participate in the experiment. The subject wore the motion capture glove in the left hand and the wrist rehabilitation robot in the right hand. The right hand was in a relaxed state as the affected hand and provided some impedance, simulating the patient's muscle stiffness. The subject's left hand moved at a comfortable speed within the maximum reachable range as much as possible. The right hand followed the left hand movement driven by the exoskeleton. The joint angles were recorded, and the equivalent end position error of the *14 cm* rod was also calculated. The simultaneous angle tracking performance of all three DOF and position error is shown in Figure 9.

It can be seen that the right hand can follow the left hand well, even if there was impedance interference. The Z-axis curve (M-Angle) demonstrates that the adaptive structure can follow the hand movement well, to change the cable attachment distribution in real-time and ensure cable tension. The passive training control algorithm can be implemented well. The cable tension curve and the parasitic force curve during the training were also recorded. It can be seen that for passive training, the required cable tension was small, and the parasitic force did not exceed *20 N*, which can fully meet the safety and comfort training requirements. During the experiment, the cable tension controller performs well. But due to the characteristics of the MR and the friction characteristics of the Bowden cable, the actual cable tension curve had a certain continuous small jitter with the amplitude ±*0.4 N*, but did not affect tracking of tension command. In the meanwhile, CDWRR with the highest performance of the existing cable-driven robots was selected for workspace comparison. It can be seen that CDWRR cannot cover the normal wrist joint space, which will have a limitation on the setting of clinical rehabilitation tasks.

**Figure 10:**
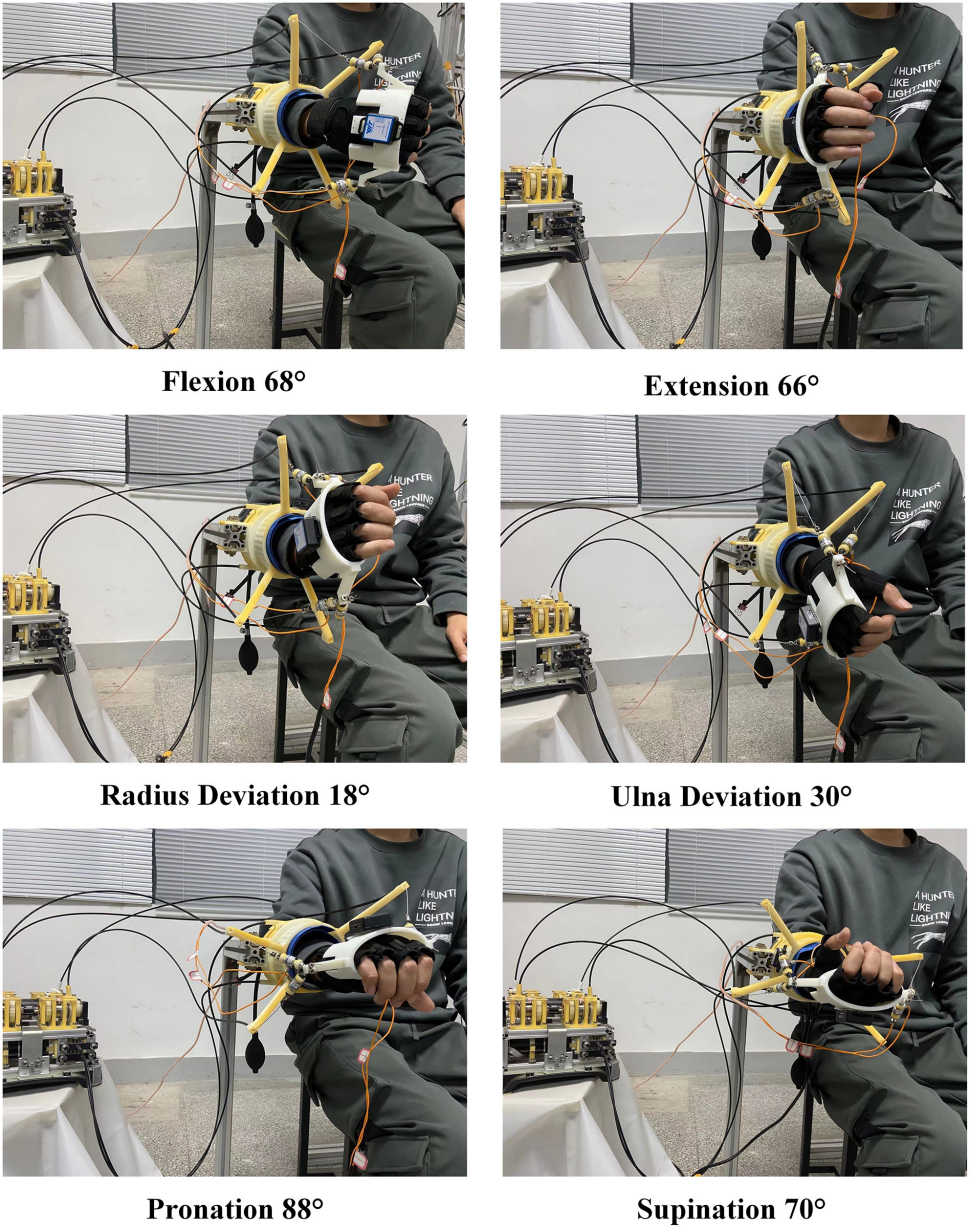
The actual workspace in experiment.

In passive training mode, the subject S1 was driven by the robot and the reachable range is demonstrated in Figure 10. The actual workspace is similar to the simulated results.

### Active Training Experiment

**Figure 11:**
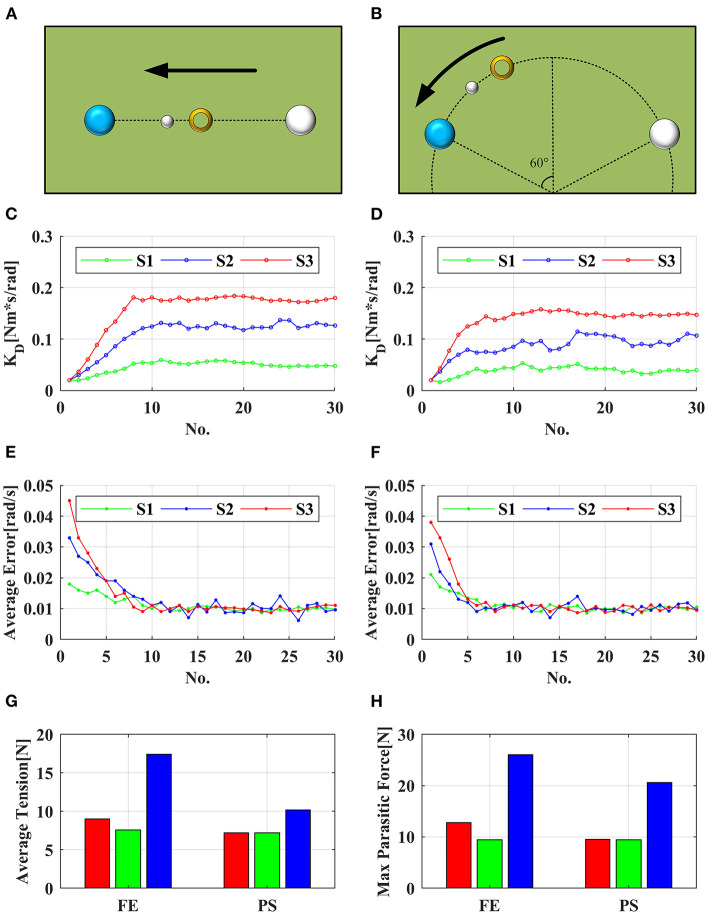
**(A,C,E)** show the visual interface, adaptive *K*_*D*_ and average error for FE experiment, respectively; **(B,D,F)** show the visual interface, adaptive *K*_*D*_ and average error for PS experiment, respectively; **(G,H)** demonstrate the average tension and max parasitic force in task no. 15–30 of S3, respectively. The red bar is the average value of SER-WRE in the large range, the green bar is its average value in the small range, and the blue bar is the average value of CDWRR in the small range.

A healthy subject S1 (26 years old, male) and two stroke patients S2 (45 years old, male, Fugl-Meyer Arm score = 52) and S3 (51 years old, male, Fugl-Meyer Arm score = 20) participated in the experiment to verify the performance of the active training. In the experiment, the subject wore the robot on the affected side, and performed FE (−50°~ + 50°) movement for the first experiment and PS (−60°~ + 60°) movement for the second experiment according to the visual indication of the display. As shown in Figures 11A,B, the blue circle is the target, the small white circle is the guide cursor, and the yellow ring is the current position. When one DOF training was performed, the feedback gain *K*_*D*_ of the other two DOFs was set high (0.5 Nm^*^s/rad) to constrain their movement. The subjects conducted pre-experiments before the formal experiment to familiarize themselves with the operation of the system. Each task was about *5 s*, and one experiment consisted of 30 tasks. *r* and *K*_*D*_ of each task were recorded, and the target error was set as *0.01 rad/s*. CDWRR was selected to compare the tension efficiency with SEU-WRE. FE (−30°~ + 30°) and PS (−10°~ + 50°) were defined as the small range, and FE (−50°~+50°) and PS (−60°~+60°) were defined as the large range. Due to the large movement range exceeded its maximum range, the average cable tension and parasitic force of CDWRR in small range were calculated for comparison. In the meanwhile, the data of SEU-WRE in small and large range were calculated, respectively.

It can be seen from the results that with the assistance of this robot, no matter the health or the patient reached the set error target after several tasks, and the error fluctuated around the target value. *K*_*D*_ was continuously adjusted according to the subjects' performance. Since the set error target was pretty small, even healthy subjects must be assisted to reach the target. It can be seen from Figure 11 that three subjects showed similar fluctuations in the FE and PS experiments. The assistance for S2 was smaller than for S3, but the assistance fluctuation was larger. It was because that S2 had good movement ability but it was unstable. Especially, S2 had poor movement accuray, so that the robot needed to provide more assistance to help him correct the wrong movement. The ability of S3 was weak, so the required assistance was high, but the overall change was stable.

For comparison of the tension efficiency, in the experiment of S3, the average value of *r*, cable tension, and parasitic force in the small range and large range of tasks 15–30 were recorded, respectively. In these 16 tasks, the average assistive torque provided by the robot did not show the siginificant difference in both large and small range. In FE, the average tension was lower in the small range than in the large range, that was, in the small movement range, the tension efficiency was higher. Comparing with CDWRR, it was obvious that SEU-WRE can provide higher tension efficiency and lower parasitic force. In PS, the average cable tension was similar in the small and large range. Because of the rotating compensation mechanism, PS movement did not change the configuration of the cables, and SEU-WRE also provided higher efficiency compared with CDWRR. In the experiment, all subjects indicated that they did not feel the abnormal effect of the parasitic force. The therapist believed that this robot is simple to operate without adjustment for individual differences and it has enough DOFs and workspace to meet the requirements of a variety of rehabilitaion training tasks.

### Safety and Comfort Experiment

To verify the safety mode proposed in this paper, one healthy subject were selected for the experiment. The FE safety range was set in (−20°~ + 20°), and the resistive training was performed in this range. The controller can then be written:

(12) $$\begin{array}{l} \tau_{r}=g\left(q\right)-B_{D}\dot{q} \end{array}$$

**Figure 12:**
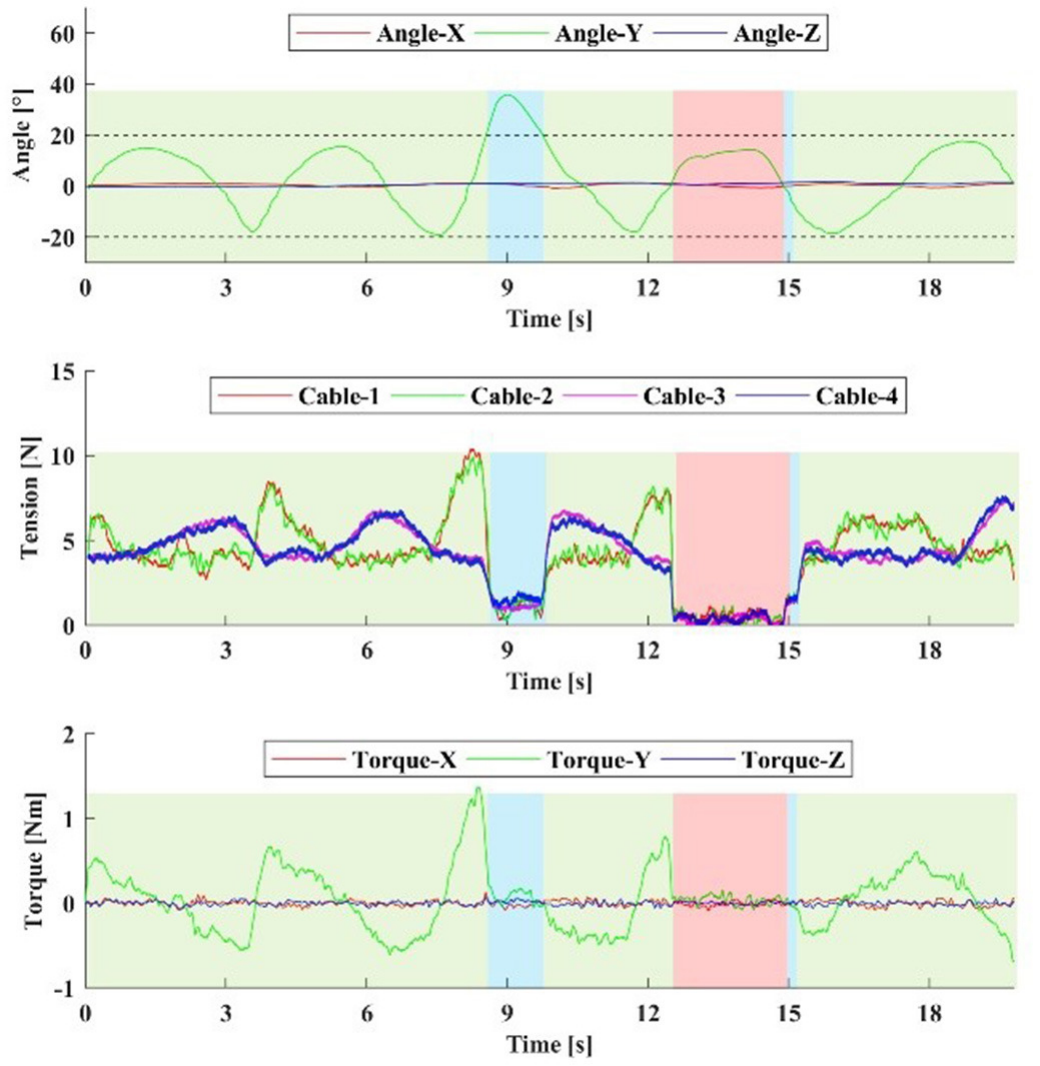
The healthy subject performs a safety mode test. The damping coefficient *B*_*D*_ is 1 Nm·s/rad. The subject actively exceeds the safe range near the 9th s, and then returns to the safe range. Near the 12th s, the drive system is powered off and the robot enters Safety Mode II. Then, the drive system is powered on near the 15th s, the robot returns to Normal Work Mode. The blue and red regions are Safe Mode I and Safe Mode II, respectively.

***B***_***D***_ is the damping coefficient. As shown in Figure 12, when the subject exceeded the boundary, the cable tension dropped to a minimum, but the cables still remained tense. At this time, the subject's hand can move freely without obvious interference. Even if the tension sensor or IMU fails, this mode is also effective. Then the subject actively moved to the safe range, and the robot quickly changed to the Normal Work Mode to provide the required assistance. When it is controlled by the software, the tension switch is not hard. Then the drive system was powered off, and the robot entered Safe Mode II. After the cables were pulled out with a very small force, the tension of all cables became zero. The cables slacked, and the subject can move freely without any interaction with the robot. The hand ring was easy to be taken off. After powering on the drive system, the system returned to Safe Mode I and to Normal Work Mode.

## Conclusion

This paper proposed a cable-driven three-DOF wrist rehabilitation training robot with high-bandwidth force-feedback function. The addition of the Z-axis rotation adaptive mechanism increases the robot workspace and tension efficiency, which is critical to the safety and comfort of rehabilitation and force-feedback. By combining the MR clutches with the geared motor, the decoupling of the output characteristics of the motor can be realized. The power system has the advantages of good safety and high force bandwidth compared to the conventional actuator. Several experiments were carried out to verify that this robot system can provide high-bandwidth force-feedback, accurate tracking performance, and safe human-robot interaction. Compared with the previous cable-driven robots, the proposed design has the larger workspace that can cover the ADLs range, and has higher tension efficiency, which can increase the effective torque and reduce the parasitic force. At the same time, it still has the characteristic of the flexibility, low inertia, free alignment.

However, due to the unidirectional tension characteristic of the cable, although the parasitic force is reduced, it cannot be eliminated completely. If this device is used for the patient with high muscle tone, the parasitic force could be large. In addition, the initial length of the cable and the length of the limb are measured manually, and it is not convenient in the clinic. In the next work, these parameters including the wrist joint center shift can be self-identified using the length of the redunctant cables. The clinical experiments are also in progress.

## Data Availability Statement

The original contributions presented in the study are included in the article/supplementary material, further inquiries can be directed to the corresponding author.

## Ethics Statement

The studies involving human participants were reviewed and approved by Zhongda Hospital Southeast Universtiy. The patients/participants provided their written informed consent to participate in this study.

## Author Contributions

KS, AS, and HL proposed and designed the robot system including the exoskeleton and the DASA system. KS and YL developed the controller and DC designed the MR clutches. KS wrote the manuscript with the help of AS and LZ. All authors contributed to the article and approved the submitted version.

## Conflict of Interest

The authors declare that the research was conducted in the absence of any commercial or financial relationships that could be construed as a potential conflict of interest.
